# Confined acoustic line modes within a glide-symmetric waveguide

**DOI:** 10.1038/s41598-022-13782-1

**Published:** 2022-06-29

**Authors:** Daniel B. Moore, Gareth P. Ward, John D. Smith, Alastair P. Hibbins, J. Roy Sambles, Timothy A. Starkey

**Affiliations:** 1 Electromagnetic and Acoustic Materials Group, Department of Physics and Astronomy, University of Exeter, Stocker Road, Devon, EX4 4QL UK; 2 DSTL, Porton Down, Salisbury, Wiltshire, SP4 0JQ UK

**Keywords:** Acoustics, Metamaterials

## Abstract

Confined coupled acoustic line-modes supported by two parallel lines of periodic holes on opposite surfaces of a glide-symmetric waveguide have a hybrid character combining symmetric and anti-symmetric properties. These hybrid coupled acoustic line-modes have a near constant group velocity over a broad frequency range as no band gap is formed at the first Brillouin zone boundary. We show that the hybrid character of these confined modes is tuneable as a function of the spacing between the two surfaces. Further we explore how the band-gap reappears as the glide symmetry is broken.

## Introduction

Acoustic metasurfaces as a branch of the wider acoustic metamaterial area have received increasing interest for beam-steering^1–4^, tailored absorption^5–8^, active systems^9,10^ and acoustic antennas^11–13^. Typically these metasurfaces are structured planar geometries that manipulate acoustic waves through the collective interaction of resonators, membranes, space coiling elements etc. when tessellated in some periodic distribution with spacing or unit-cell dimensions smaller than the order of the wavelength. Combining these elements allows for a surface with designer effective medium properties, such as negative mass-density and refractive index utilised by flat acoustic lenses^14,15^ and impedance-matched absorbers^16,17^. Metasurfaces comprised of periodic resonators can often support non-radiative acoustic modes, termed acoustic surface waves (ASWs), localised at the interface between the metasurface and the surrounding fluid. These modes therefore allow sound to be guided on a surface over a range of frequencies and wavenumbers dictated by the surface patterning, and the resulting dispersion properties. ASWs can be formed by having resonant cavities, in our case simple ‘blind’ holes, which are close enough together so that there is near-field diffractive coupling of neighbouring resonators. On an otherwise flat surface, a periodic line of such blind holes supports an ASW that propagates along the line of holes, and which decays evanescently away from the surface due to the effective impedance condition of the rigid cavities patterning the surface^18^. The behaviour of these modes is strongly dependent on the volumetric resonances of the cavity and the unit cell periodicity. The dispersion characteristics and number of acoustic modes supported can be further tailored by, for example, the introduction of structure factor (for example the number of resonators per unit cell^19–21^) or symmetries^22,23^. Previous studies have shown that the number of supported resonances is linked to the degrees of freedom within the unit cell^24^. Additional phase resonant modes can be introduced by the structure factor^4,21,25^. Furthermore, symmetries, such as honeycomb tessellations can result in regions of strong linear dispersion and Dirac-like crossings in the band structure^26,27^. Ward *et at.*^28^ demonstrated that a simple 1D structure comprised of a line of resonant holes can control the propagation of sound using ASWs. The waveguide-like modes supported on these systems are termed acoustic line-modes (ALMs), due to the arrangement of unit cells along the sample allowing the control of sound propagation along specific surface pathways. This potential for controlling sound by ALMs makes them suitable for applications such as sensing^29^. However, the ALM dispersion, with very low group velocity and increased losses as the mode frequency approaches the upper limit arising from the Bragg diffracted standing wave, does rather limit its potential as wave amplitude attenuates as the mode propagates^28,30^.

In this study, the coupled ALMs formed by a waveguide comprised of two parallel surfaces each having a line of periodically spaced, identical holes placed opposite each other are explored. This geometry allows, through the relative longitudinal displacement, the exploration of the coupled ALMs formed between two metasurfaces with mirror and glide symmetry conditions, as well as anywhere in between these symmetric limits. Our paper is organised as follows. First the metasurface geometries will be described, with attention to the symmetry conditions that our sample geometry may provide. The allowed eigenmodes, calculated using numerical models are then evaluated, before experimental results that compare and contrast the different symmetry conditions explored; namely, mirror- and glide-symmetry waveguide configurations are presented. Finally, the effects of breaking the symmetry conditions, so the waveguides are in neither mirror or glide configuration, will be explored.

## Results

### Metasurface geometry and numerically modelled mode dispersion

**Figure 1:**
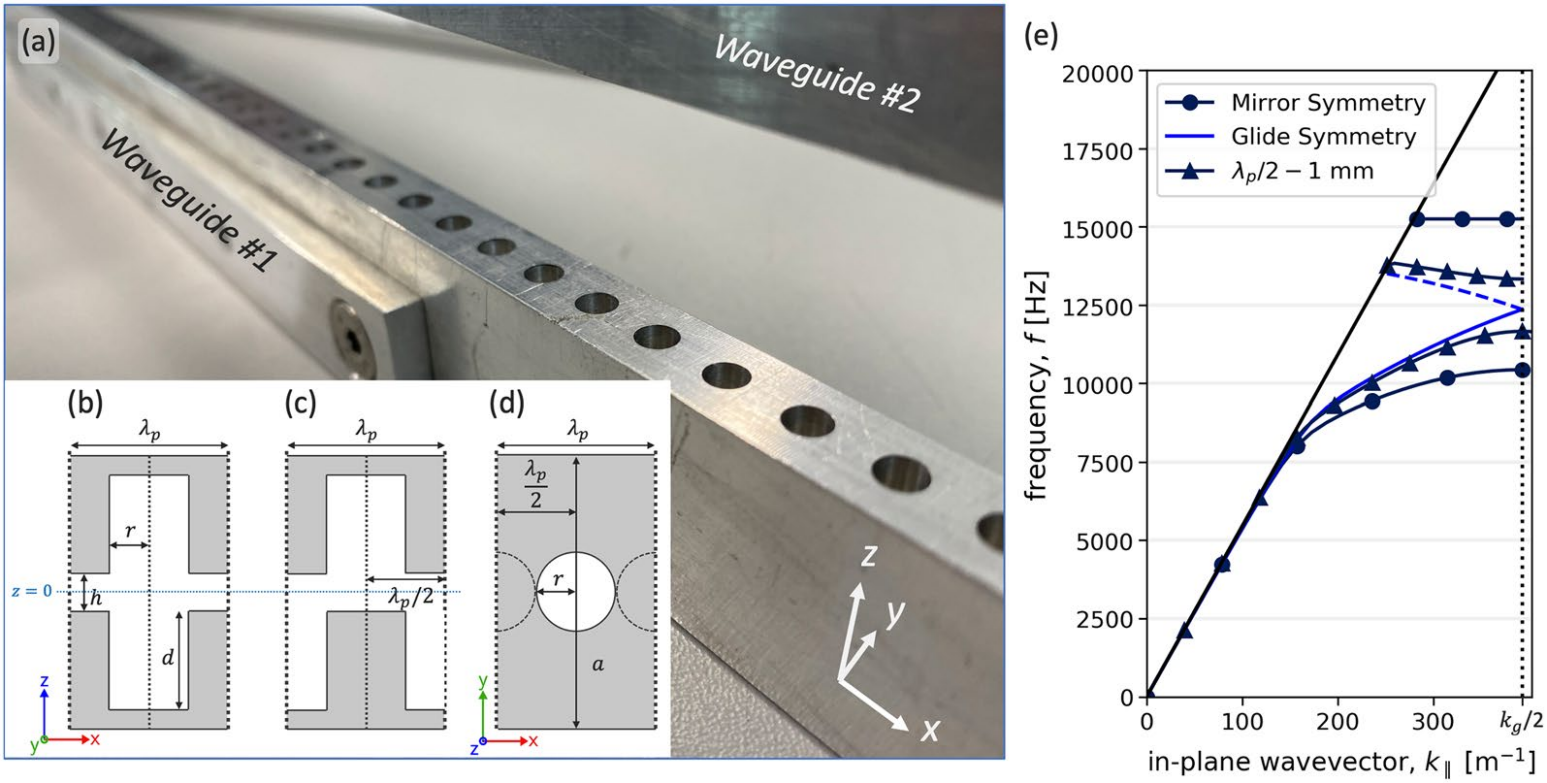
(**a,b,c,d**) Photograph and schematic diagrams of the sample shown in both mirror and glide symmetry arrangements. In (**b**,**c**,**d**), areas of grey represent aluminium, and white represents air. The sample has a periodicity $\lambda _{p}$ = 8 mm along the x axis, and is patterned with cylindrical resonators with depth *d* = 5 mm and radius *r* = 2 mm. Both surfaces have width *a* = 10 mm in the y axis. These two surfaces, whether in mirror or glide symmetry, are separated by height *h*, which may be varied. (**a**) Photograph of the aluminium sample. (**b**) Schematic showing mirror symmetry, where the patterned surfaces are perfectly mirrored normal to the z axis. (**c**) Shows glide symmetry, where the surfaces are mirrored normal to the z axis and translated along the x axis by half a unit cell period ($\lambda _{p}$/2). (**d**) A top view of the sample in glide symmetry, where the opposing surface resonators are indicated as dashed lines. (**e**) FEM calculated dispersion relations of eigenmode solutions showing the acoustic surface waves supported on the sample in mirror and glide symmetry arrangements, as well as $\lambda _p/2 - 1$ mm for surface separation *h* = 1.5 mm. Dashed lines indicate modes that have been scattered back into the 1st BZB. The solid black line represents the sound-line ($k_{0}$) with vertical dotted line indicating the first BZB at $k_{\parallel }$ = $k_{g}/2$.

**Figure 2:**
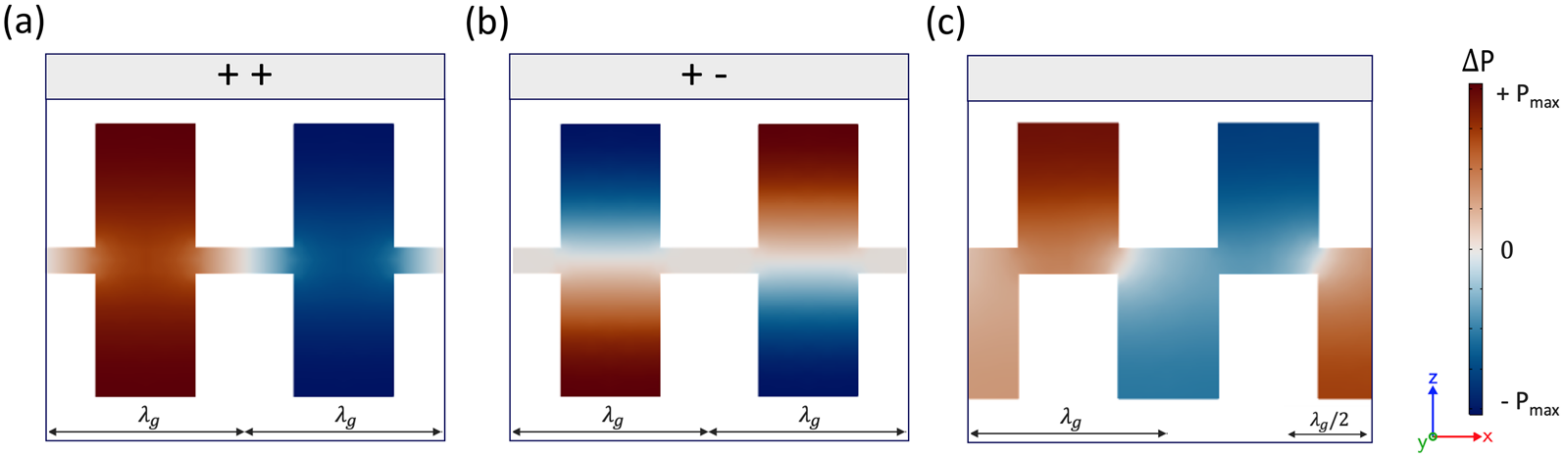
(**a,b,c**) Instantaneous pressure fields $\Delta$P (with $\Delta P=\left| {P-P_0}\right|$, where $P_0$ is the background pressure field) of eigenmodes calculated using loss-inclusive FEM simulations. Plots show a xz-slice through the centre of the sample (*a*/2) along the direction of propagation (x axis). White areas indicate the acoustically rigid aluminium substrate, separated in the z axis. (**a**) The fundamental, or symmetric mode (labelled ++) of the mirror symmetry system at the 1st BZB ($k_{\parallel }$ = $\pi /\lambda _{p}$). (**b**) The anti-symmetric mode (labelled +-) of the mirror symmetry system taken at the 1st BZB ($k_{\parallel }$ = $\pi /\lambda _{p}$). (**c**) The degenerate mode of the glide symmetry system at $k_{\parallel }$ = 423 m$^{-1}$.

The simplest geometry to explore is a single row of holes in an isolated surface: consider holes with radius *r*, and depth *d*, periodically spaced by $\lambda _p$. The low energy mode supported by this structure is the fundamental resonance, given approximately by $f_{res}=c/4(d+\Delta L)$, where *c* represents the adiabatic speed of sound in air and $\Delta L$ represents the end correction for a single opening ($\Delta L = \frac{8r}{3 \pi } \approx 0.8 r$)^31,32^. The lowest frequency band is indistinguishable from the free-space wave or sound-line $k_\parallel = k_0$ with $k_0=2\pi f/c$ for small values of $k_\parallel$, see Fig. 1e. As $k_\parallel$ increases, the mode progressively disperses away from the sound-line, becoming more strongly bound to the surface. Further increasing $k_\parallel$ sees the mode meet the first Brillouin zone boundary (BZB) (where $k_{\parallel }$ = $k_{g}/2$ with $k_g=2\pi /\lambda _p$) with zero group velocity, at which the wavelength along the structure is twice the periodicity of the lattice. The addition of another surface supporting an identical ALM propagating parallel to the first one, with the holes in the two surfaces lying exactly opposite each other, giving mirror symmetry, is shown as a schematic in Fig. 1b and as instantaneous pressure fields in Fig. 2a,b. In this case there are two resonators per unit cell so two distinct modes are supported, which are split at the 1st BZB into an upper and lower energy pair of standing waves as shown in Fig. 1e. The low energy branch has the pressure fields in opposite cavities oscillating in phase, while the higher energy branch has the pressure fields in opposite cavities oscillating in anti-phase, with zero amplitude along the plane of symmetry lying at the mid-plane between the two surfaces^33^.

A second high symmetry arrangement is that of glide symmetry. Glide symmetry is a modification of mirror symmetry, where a periodic system is mirrored about a defined mirror plane and an offset of half a unit cell (${\lambda _{p}}/2$) is applied to one axis^22,23,34,35^. Figure 1b–c shows schematics for both mirror and glide symmetries. Glide symmetry effectively halves the periodicity of the structure, doubling the size of each Brillouin zone. This is evident in the band structure of the system’s eigenmodes, where the degenerate mode is band folded back into the first Brillouin zone^29,36^, shown in Fig. 1e as a dashed line. In a system with glide symmetry the symmetric/anti-symmetric pair of modes supported by the surface are fully degenerate at the BZB and the band gap closes. The mode does not form a standing wave state and its dispersion continues through the 1st BZB with finite gradient into the second Brillouin zone^22,29,37^. The pressure fields of the mode at the 1st BZB for the glide symmetry case is illustrated in Fig. 2c. This is in contrast to the mirror symmetric system where the pressure fields are symmetric about the z axis for the lower energy mode of the band gap, and anti-symmetric for the upper energy mode. Furthermore, this degenerate mode exhibits a non-zero, near constant group velocity at the first BZB, giving a 3 kHz frequency band, between $\approx$ 10 to 13 kHz, over which very slow sound propagates, as shown by the reduced curvature of the mode in Fig. 1e. One may of course, with this two surface structure, explore any offset of the upper surface relative to the lower in the propagation direction. One finds that as the displacement is slowly deviated from $\lambda _{p}/2$ (the glide condition) the band gap at the BZB reappears and the group velocity at the BZB returns to zero, as shown in Fig. 1e.

### Experimental results

**Figure 3:**
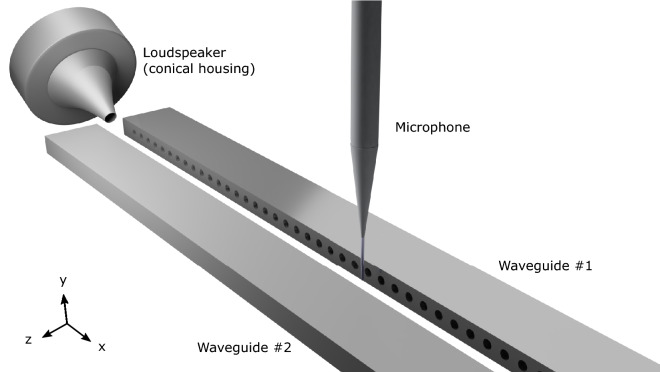
Render illustrating the acoustic measurement experiment, the loudspeaker mounted within the conical housing is shown positioned at a grazing incident angle to the sample and the microphone positioned within the formed cavity between the waveguides.

**Figure 4:**
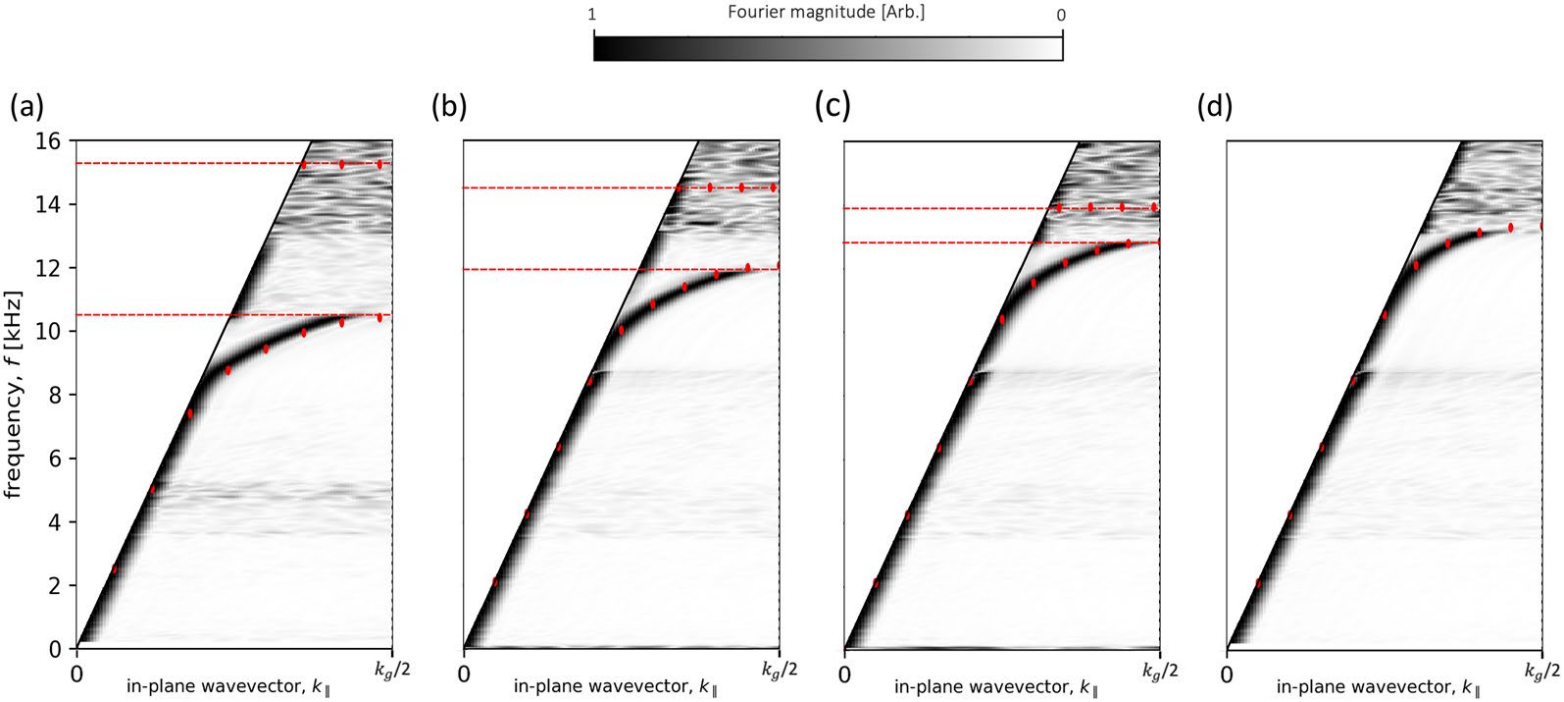
Experimental dispersion curves for the sample in mirror symmetry, at different separations. (**a**) *h* = 1.5 mm , (**b**) *h* = 3 mm, (**c**) *h* = 5 mm and (**d**) *h* = 10 mm. Absolute Fourier amplitude is shown as a function of frequency and in-plane wavevector $k_{\parallel }$ with the colour representing the Fourier amplitude. Data is normalised using slices in frequency. Solid lines represent the sound-line ($k_{0}$) with vertical dotted lines indicating the first BZB at $k_{\parallel }$ = $k_{g}/2$. Red points show eigenfrequency predictions from loss-inclusive numerical models. Red dashed lines represent formed bandgaps.

**Figure 5:**
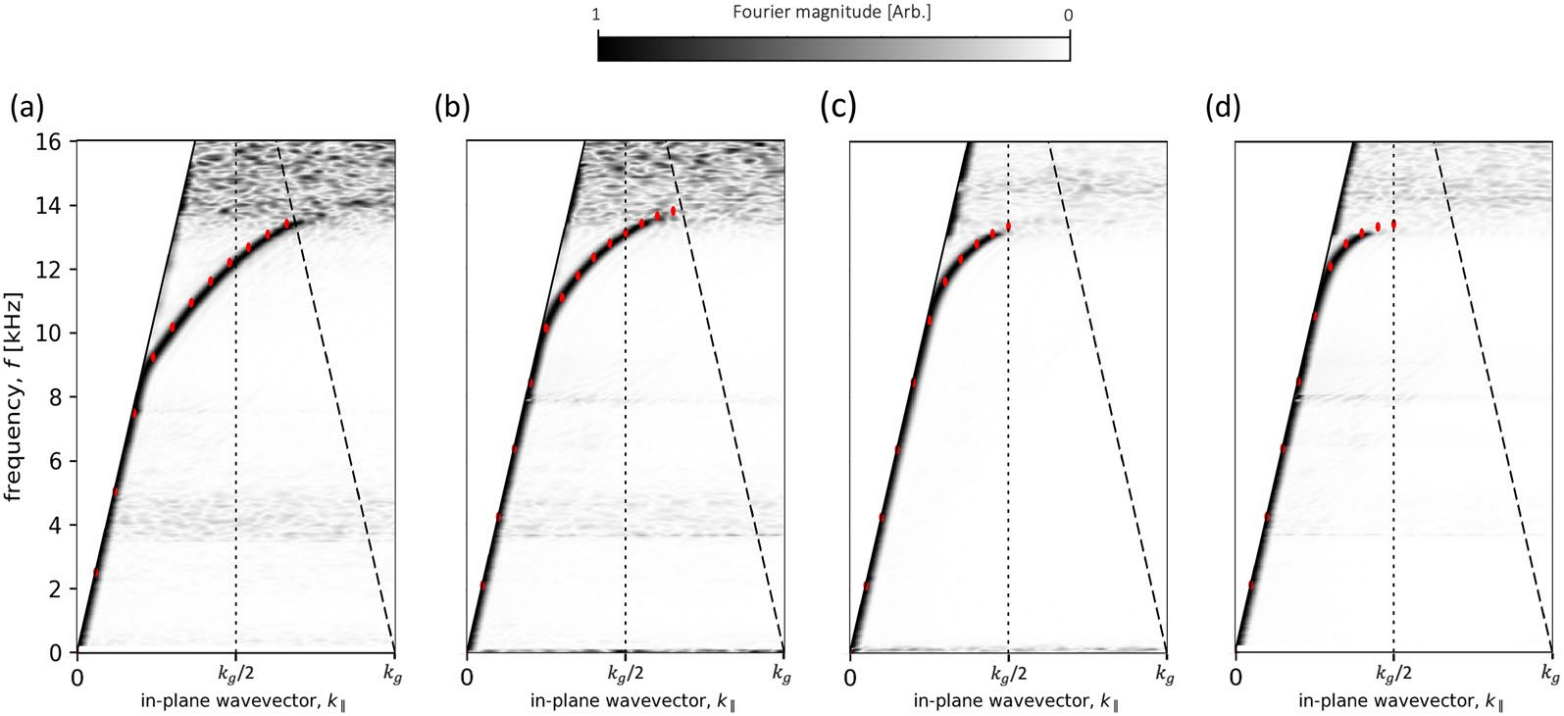
Dispersion diagrams calculated from experimental data for the sample in the glide-symmetric arrangement, at different separations along the z axis. (**a**) *h* = 1.5 mm, (**b**) *h* = 3 mm, (**c**) *h* = 5 mm and (**d**) *h* = 10 mm. Absolute Fourier amplitude is shown as a function of frequency and in-plane wavevector $k_{\parallel }$ with the colour representing the Fourier amplitude. Data is normalised using slices in frequency. Solid black lines represent the sound-line ($k_{0}$) and dashed black lines the diffracted sound-lines with vertical dotted lines indicating the first BZB at $k_{\parallel }$ = $k_{g}/2$. Red points show eigenfrequency predictions from a loss-inclusive numerical model.

**Figure 6:**
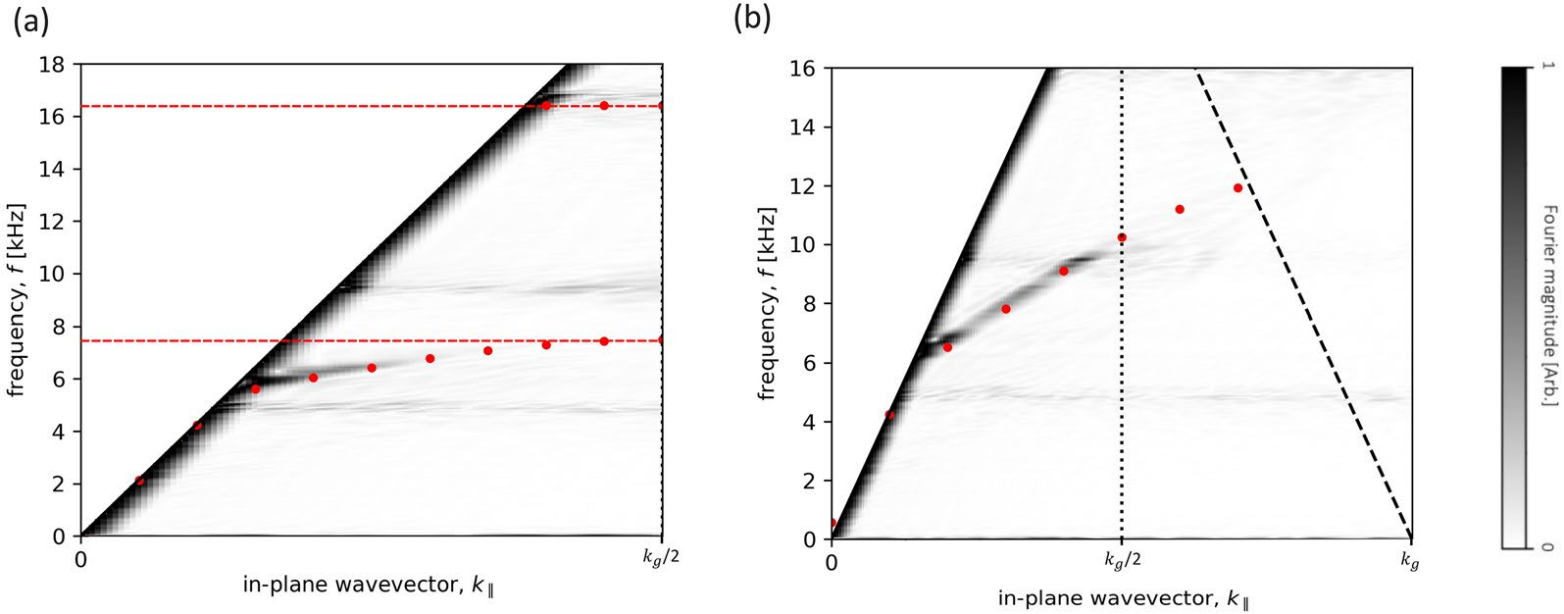
Experimental dispersion for the sample in both (**a**) mirror symmetry and (**b**) glide symmetry arrangements, with surface separation, *h* = 0.5 mm. Absolute Fourier amplitude is shown as a function of frequency and in-plane wavevector $k_{\parallel }$ with the colour representing the Fourier amplitude. Data has been normalised using slices in frequency. Solid black lines represent the sound-line ($k_{0}$) and dashed black line the diffracted sound-line with vertical dotted lines indicating the first BZB at $k_{\parallel }$ = $k_{g}/2$. Red points show eigenfrequency predictions from a loss-inclusive numerical model. Red dashed lines represent the formed bandgap.

**Figure 7:**
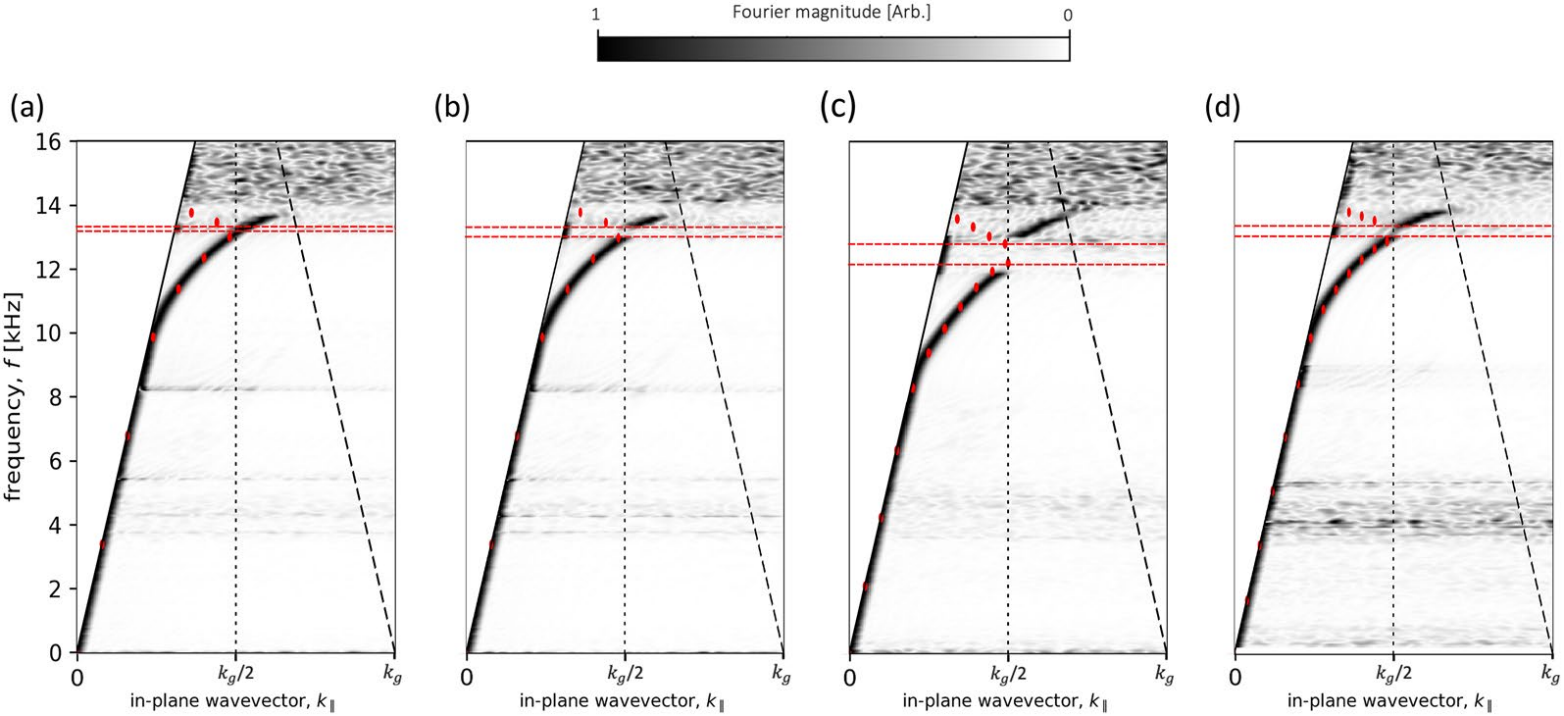
Experimental dispersion diagrams for the sample at a range of translations conditions using a separation, *h* = 3 mm. (**a**) $\lambda _p/2 - 0.05$ mm x axis translation, (**b**) $\lambda _p/2 - 0.15$ mm x axis translation, (**c**) $\lambda _p/2 - 0.35$ mm x axis translation and (**d**) $\lambda _p/2 - 0.5$ mm x axis translation. Absolute Fourier amplitude is shown as a function of frequency and in-plane wavevector $k_{\parallel }$ with the colour representing the Fourier amplitude. Data is normalised using slices in frequency. Solid black lines represent the sound-line ($k_{0}$), dashed black lines the diffracted sound-line with vertical dotted lines indicating the first BZB at $k_\parallel =k_g/2$. Red points show eigenfrequency predictions from a loss-inclusive numerical model. Red dashed lines represent formed bandgaps. The experimental dispersions show a bandgap opening as the x axis translation is increased. However, with (**a**) being such a small displacement, the fit is not exact and the bandgap is not as pronounced. A bandgap is present, indicated by the intensity shift to the sound-line between the two predicted eigenmode solutions.

To verify the numerical predictions we experimentally characterise the ASWs confined within the waveguide in mirror and glide symmetry arrangements. The model dispersion relation as a function of the in-plane wavenumber ($k_\parallel = \sqrt{k_x ^2 + k_y ^2}$, where $k_{x\mid y}$ is the x$\mid$y axis component) was obtained using the Finite Element Method (FEM) eigenmode numerical simulations (see "Methods"). The dispersion was experimentally obtained by mapping the acoustic pressure field within the formed cavity between the surfaces; a 26 mm tweeter mounted inside a conical housing excited with a Gaussian envelope pulse was positioned at a grazing angle to the sample and the local pressure field measured by a microphone positioned within the waveguide attached to an xy translation stage (for an illustration see Fig. 3 and for more details see "Methods"). The overlap of the frequency- and wavevector wavefunction of both the source and surface mode determines the extent to which the ALM is excited. By scanning the microphone along the waveguide, a 1D map of the time-dependent signal as a function of position is collected. From this, the full dispersion relation can be produced through temporal and spatial Fourier analysis. The dispersion relation data presented in the following sections has been normalised to its maximum value with respect to frequency (Figs. 4, 5, 6, 7). Data within the radiative domain was removed prior to normalisation. The noise visible in Figs. 4, 5, 7 from $\approx$ 14 kHz is an artifact of the measurement data being normalised with respect to frequency. Direct signal from the source was minimal due to the microphone being placed within the formed cavity between the surfaces. In the absence of a surface wave signal or direct radiation, the noise floor is normalised against itself, resulting in random noise.

#### Mirror symmetry arrangement

Figure 4a–d shows the measured dispersion of the modes of the sample, arranged so that the two surfaces are in mirror symmetry for a range of surface separations *h*, with the results from a loss-inclusive FEM model overlaid as red points. The results in Fig. 4a–d show the asymptotic frequency (the frequency at the BZB the mode is approaching), decreases in frequency as the surface separation is decreased. At first sight this is perhaps unexpected, as the gap between the surfaces is reduced one may suppose that the end effects, which normally reduce the resonant frequency of the holes from the simple quarter wavelength condition, will diminish and the frequency will rise. For a separation *h* = 10 mm (Fig. 4d) the two surfaces are uncoupled. With neither surface “knowing” the other’s periodicity, the 1st BZB no longer sits at $k_g/2$ as previously defined, and the mode localised at other surface passes through it. As the surfaces are brought closer together, the end-effects of the opposing resonators begin to overlap and, instead of decreasing, they constructively interfere and the combination starts to resemble single resonators with an increased length, becoming twice the length of a single cavity as the gap goes to zero, and closed at both ends. Conversely, the asymptotic limit of the anti-symmetric mode increases with coupling strength due to the zero-pressure midway between the $\pm ~p_{max}$ states of the resonators tightly confining the resonance within the cavities, reducing the end-effects and increasing the frequency up to the quarter wavelength condition. Furthermore, once the surfaces are uncoupled the anti-symmetric mode can no longer exist^31,32^. As an illustration of the agreement with the model data, we estimate in the *h* = 1.5 mm case, at $\approx$10 kHz a reduction in the velocity of sound in air by a factor of 11. The FEM model predicts a reduction in the speed of sound by a factor of 14, suggesting close agreement between the FEM model and the experimental data. The predicted higher energy anti-symmetric mode has not been detected in the experiment. This is partly because the excitation of this mode is problematic as it requires zero pressure halfway between the two surfaces. Further the FEM modelling results suggest the anti-symmetric mode is rather broad, with almost zero group velocity (see the high frequency mode for the mirror symmetry arrangement in Fig. 1e). Thus, even if excited, it would not propagate far from the source.

#### Glide symmetry arrangement

Figure 5a–d show the experimentally determined dispersion of the glide-symmetric sample for a range of separation values, with the results from a loss-inclusive FEM model overlaid as red points.

The dispersion shows a low frequency ALM which differs from that formed in the mirror symmetric case as at the BZB there is only a single mode with no bandgap. Further this mode appears to be dispersing towards a different asymptotic frequency to the mirror-symmetric counterpart. Another feature of the coupled ALMs shown in Fig. 5 is the significant frequency range over which there is a near constant group velocity. For separation *h* = 1.5 mm, the group velocity ($v_g$) is constant between $\approx$ 9.5 and 13.5 kHz, with a coefficient of determination ($R^2$) of 0.99. The group velocity was found by calculating the gradient of the model data between $\approx$ 9.5 and 13.5 kHz and linear regression used to quantify its variance from a linear fit. At $\approx$ 13 kHz the speed of sound in air has been reduced by a factor of 7. Furthermore, the FEM model also predicts a reduction in the speed of sound by a factor of 7, showing good agreement between the FEM model and the experimental data. In addition, the coupled ALM in the glide symmetric case is measurable for wavevectors well beyond the first BZB. Data for a range of separations show that the range of $k_\parallel$ values over which data is obtained substantially reduces as the surface separation increases and the coupling of the two ALMs weakens. Figure 4 shows that the asymptotic frequency at the first BZB of the mirror-symmetric sample changes significantly as a function of the sample separation, whereas, Fig. 5 indicates that the asymptotic frequency for the glide-symmetric sample is largely independent of sample separation. This is not surprising since the cavities are now no longer opposite each other, and the offset by $\lambda _p/2$ prevents the end-effects of adjacent resonators from interacting as in the mirror symmetric sample.

#### Effect of small separation with both mirror and glide symmetries

Additional measurements were taken with the sample arranged in both mirror and glide symmetries with a surface separation, *h* = 0.5 mm. The microphone was positioned 0.5 mm outside the formed cavity for these measurements due to the diameter of the microphone exceeding the width of the formed waveguide. The calculated dispersion plots in Fig. 6 show a reduced asymptotic frequency for the mirror symmetry sample compared to the previously discussed results. Note also the increased intensity of the sound-line compared to previous results. This is due to the probe being positioned outside the cavity and detecting more of the free-space wave ($k_{0}$). One would expect that losses due to viscosity arising from the no-slip condition at the walls of the sample would become more pronounced at reduced separation values. When the separation between the surfaces is reduced, thermal-viscous boundary layers occupy an increased percentage of the waveguide, strongly affecting the propagation of sound through the structure. At a separation, *h* = 0.5 mm the viscous boundary layers ($\delta _v$) within the sample are calculated to be $\approx ~28~\mu$m at 12 kHz^38^, occupying $\approx ~6\%$ of the separation and thereby altering the effective speed of sound within the waveguide^31,39^.

Results in Fig. 6 show the modes deviating from the sound-line at a lower frequency compared to the wider separations discussed previously. The width of the mode in the glide-symmetric case appears broader compare to previous results, indicating that the losses within the waveguide have increased. For the glide-symmetric sample the mode displays a constant group velocity over a broader frequency range ($\approx$ 6 to 11 kHz, with $R^2= 0.83$). In the case of the mirror symmetry sample, the formed bandgap occupies a greater frequency range compared to wider separation values, and demonstrated a greater reduction of the speed of sound (a factor of 22 at $\approx ~7.5$ kHz).

#### Non-glide symmetry translation condition

All the above results use either mirror or glide symmetry translation conditions; the plots in Fig. 7 show the experimentally determined dispersion of the sample taken for other translation values ($\Delta _x$). Results from a loss-inclusive FEM model are overlaid as red points. The ALMs supported in the glide-symmetric system are formed from the symmetric and anti-symmetric modes forming a degenerate pair. As this characteristic is reliant on the $\lambda _p/2$ translation, one would expect that when the translation is broken by any arbitrary amount, the modes no longer form a degenerate pair, resulting in two modes separated by a bandgap at the BZB. The measured dispersion relations in Fig. 7 demonstrate that a deviation from glide-symmetry of 0.05 mm is sufficient for a bandgap to form. However, a longitudinal displacement of 0.05 mm (Fig. 7a) is such a so small translation (0.6% of the unit cell length), the fit is not exact and the bandgap is not as pronounced. For the $\lambda _p/2 - 0.05$ mm case the bandgap is indirectly observed, by the intensity shift to the sound-line between the two predicted eigenmode solutions (see Fig. 7a).

## Conclusions

Experimental data has been obtained for the dispersion of the coupled ALMs formed between two identical surfaces both as the separation between them is changed and as the displacement of one relative to the other along the propagation direction is altered. All the data is compared with FEM models that include viscous damping. For the mirror symmetry (zero x axis displacement) reducing the separation between the two metasurfaces somewhat surprisingly reduces the asymptotic frequency as the acoustic fields in the aligned cavities extend into each other and thereby lower the resonant frequency. For the very thin surface separation, *h* = 0.5 mm the coupled mode is further increased in $k_\parallel$ for a given frequency because of the decreased sound velocity arising from viscous effects within the gap. For the glide symmetry case ($\lambda _p/2$ x axis displacement) the mode has a finite group velocity at the BZB and it is found to extend in $k_x$ well beyond the BZB. With the smallest separation, *h* = 0.5 mm the mode extends all the way to the second BZB where a band gap and zero group velocity is found. Finally, we have experimentally characterised the supported modes for x axis displacements different to the special symmetry cases. For an x axis displacement away from glide symmetry of as little as 0.05 mm a measurable band gap appears at the BZB. This study provides a demonstration of controlling sound within a waveguide using symmetry and translation conditions to produce tunable dispersion, which could be useful in designing acoustic devices for sensing and tailored sound absorption applications.

## Methods

### Numerical simulation

Surface mode dispersions were calculated with COMSOL Multiphysics (version 5.6)^40^ using the Finite Element Method. The dispersion relations shown are eigenmodes of a unit cell (see Fig. 1) with Floquet-periodic boundaries to represent an infinite sample. The thermal and viscous losses have been accounted for in the model, resulting in a reduction in frequency of the modes at the Brillouin zone boundary of approximately 50 Hz, compared to a loss free system. The model assumes individual resonators are cylindrical and perforate an acoustically rigid surface.

### Sample manufacture

The sample is comprised of two surfaces of aluminium, each with length *L* = 640 mm, a periodicity $\lambda _{p}$ = 8 mm along the x axis (80 unit cells total), and patterned with cylindrical resonators with depth *d* = 5 mm and radius *r* = 2 mm, see Fig. 1a–d for schematic and photo.

### Acoustic measurements

The dispersion for each separation value was characterised by measuring the acoustic near field using a probe microphone mounted on a motorised xy scanning stage. The sample was excited by a Scanspeak R3004/602000 26 mm tweeter mounted within a conical attachment with a 3 mm exit diameter and positioned at an angle to the surface to maximise diffractive coupling to the surface wave. To minimise signal from direct radiation, the needle microphone (Brüel & Kjær Probe Microphone type 4182) was normally positioned within the centre of the formed cavity provided the spacing was sufficient. The cavity was scanned at a resolution of 0.29 mm for a scan length of 400 mm (x axis). The sample was excited by a 20 kHz Gaussian envelope pulse (broadband) at each microphone position. An average was taken over three measurements at each spatial position to improve signal-to-noise.

For the reduced separation measurements (Fig. 6) when the probe diameter exceeded the separation the tip of the needle microphone was positioned 0.5 mm outside the formed cavity.

### Experimental data analysis

The dispersion was characterised by performing a Fast Fourier Transform (FFT) on the measurement data to obtain the Fourier amplitude as a function of wavevector. All dispersion relation data was processed using Hamming window functions in real space and zero padded both spatially and temporally by a factor of 4, before being Fourier transformed. The calculated dispersion data was normalised by sampling the data in the frequency axis and dividing each slice against its maximum value. Data in the radiative domain was removed prior to normalisation to prevent the increased amplitude of the free-space wave relative to the surface wave from biasing the normalisation. All data processing was done using the Numpy^41^, SciPy^42^ and Scikit-learn^43^ packages in Python 3.7.6^44,45^.

The noise visible in Figs. 4, 5, 7 from $\approx$ 14 kHz is an artifact of the measurement data being normalised with respect to frequency. The noise is caused by the mode having zero group velocity resulting in no net power flow within the sample beyond the asymptotic limit. Direct signal from the source was minimal due to the microphone being placed within the formed cavity between the surfaces. In the absence of a surface wave signal or direct radiation, the result is random due to the noise floor being normalised against itself. The horizontal features in Figs. 4, 5, 7 at $\approx$ 4 and 9 kHz are the result of nulls in the source spectra caused by internal reflections within the conical attachment, which are amplified during the normalisation.

## Data Availability

All data created during this research are openly available from the University of Exeter institutional repository at https://ore.exeter.ac.uk/.
